# Case Report: Severe hemophilia B patient with inhibitor and anaphylaxis reaction to FIX, successfully managed with concizumab prophylaxis therapy

**DOI:** 10.3389/fped.2025.1576821

**Published:** 2025-04-28

**Authors:** Elisa Bonetti, Maria Pia Esposto, Ada Zaccaron, Chiara Guardo, Giulia Caddeo, Matteo Chinello, Rita Balter, Vincenza Pezzella, Virginia Vitale, Simone Cesaro

**Affiliations:** Department of Mother and Child, Paediatric Haematology Oncology, Azienda Ospedaliera Universitaria Integrata Verona, Verona, Italy

**Keywords:** hemophilia B, factor IX inhibitors, concizumab, tissue factor pathway inhibitor (TFPI), anaphylaxis to factor IX

## Abstract

**Background:**

Hemophilia B is a rare X-linked disorder characterized by factor IX (FIX) deficiency, leading to spontaneous bleeding episodes predominantly affecting joints and muscles. Severe cases with FIX activity levels below 1% can develop inhibitors, rendering replacement therapy ineffective and posing additional challenges such as allergic or anaphylactic reactions to FIX infusions. Novel non-factor therapies, including concizumab, offer alternative strategies by targeting tissue factor pathway inhibitor (TFPI), a key regulator of coagulation. Concizumab restores thrombin generation and hemostasis, bypassing the need for FIX. Administered subcutaneously, it reduces treatment burden while enhancing adherence and quality of life.

**Case presentation:**

We report a pediatric case of severe hemophilia B with inhibitors and recurrent anaphylactic reactions to FIX therapy, and transitioned to concizumab therapy. Initial treatment included FIX replacement but repeated allergic reactions necessitated bypassing therapy such as recombinant activated factor VII (rFVIIa) and later concizumab. Following the initiation of concizumab, the patient experienced significant reductions in bleeding episodes, improved joint health scores, and decreased reliance on rFVIIa, with no hospitalizations or severe adverse events over four years.

**Discussion and conclusion:**

This case highlights concizumab's transformative role in managing hemophilia B with inhibitors, demonstrating its potential to address unmet clinical needs and improve outcomes, as corroborated by pivotal clinical trials. Comprehensive multidisciplinary care remains essential for optimizing long-term results.

## 1 Introduction

Hemophilia B is a rare X-linked bleeding disorder caused by a deficiency in clotting factor IX (FIX). Patients with severe hemophilia B, defined by FIX activity levels below 1%, often experience recurrent spontaneous bleeding episodes that predominantly affect joints and muscles. These bleeds lead to progressive joint damage, chronic pain, and diminished quality of life if untreated (1–4).

Standard treatment involves prophylactic intravenous administration of FIX concentrates, which has significantly improved outcomes by reducing bleeding episodes and minimizing long-term complications (1, 3, 4). However, approximately 1%–3% of patients with hemophilia B develop inhibitory antibodies against FIX, rendering replacement therapy ineffective (2, 3, 5). These inhibitors often arise in the context of severe gene mutations, such as large deletions or nonsense mutations, and are associated with a higher risk of severe allergic or anaphylactic reactions to FIX administration and nephrotic syndrome (1, 5–7). This clinical challenge necessitates alternative therapeutic strategies (4–8).

The advent of non-factor therapies such as concizumab offers a promising alternative for patients with hemophilia B, particularly those with inhibitors (9). Concizumab is a humanized monoclonal IgG4 antibody targeting tissue factor pathway inhibitor (TFPI), which plays a pivotal role in downregulating the initiation phase of coagulation. TFPI inhibits the TF/FVIIa-mediated activation of FX, forming inhibitory complexes that block thrombin generation (3, 4, 8, 10). By neutralizing TFPI, concizumab restores thrombin generation, bypassing the need for FIX and enabling effective hemostasis (3, 8, 11).

Concizumab also addresses several limitations of traditional therapies. Administered subcutaneously using a convenient pen injector, it alleviates the burden of frequent intravenous infusions and associated complications, such as difficult venous access or other complications due to central venous access (4, 8). Clinical trials, including the EXPLORER research program, have demonstrated the efficacy of concizumab in significantly reducing annualized bleeding rates (ABRs) in patients with hemophilia B with inhibitors. These studies also highlight an overall therapy's safety profile and minimal adverse reactions (4, 5, 12). Furthermore, concizumab has been associated with improvements in patient-reported outcomes, such as reduced treatment burden and enhanced health-related quality of life (HRQoL) (4).

This report discusses the clinical course and outcomes of a pediatric patient with severe hemophilia B and inhibitors who transitioned to concizumab therapy after experiencing recurrent anaphylactic reactions to FIX, highlighting its potential to address unmet needs in this challenging patient population. Written informed consent was obtained for the publication of these case report.

## 2 Case description

A male patient was diagnosed with severe hemophilia B at 9 months of age following an evaluation for spontaneous subcutaneous hematomas. Laboratory tests revealed a factor IX (FIX) activity of less than 1%, and genetic analysis identified a point mutation in exon H of the FIX gene, leading to a premature stop codon (TGA).

At 10 months of age, the patient underwent a T9-L2 laminectomy due to a spontaneous spinal epidural hematoma. During this hospitalization, he received his first dose of recombinant FIX (rFIX; BeneFIX, Wyeth Europa Ltd, Taplow, United Kingdom) together with dexamethasone.

After this episode, he started the secondary prophylaxis with r FIX.

However, after the 31st infusion, he experienced a severe anaphylactic reaction, presenting with coughing, respiratory distress, and peripheral cyanosis. The infusion was immediately discontinued, and the patient was treated with hydrocortisone and intravenous fluids. At that time, his inhibitor titer was measured at 0.8 Bethesda units (BU), peaking at 1.6 BU one week later.

For the next six years, the patient was managed on an on-demand regimen with recombinant activated factor VII (rFVIIa; NovoSeven, Novo Nordisk, Bagsværd, Denmark). During episodes of right elbow hemarthrosis, which became his target joint, activated prothrombin complex concentrate (APCC; FEIBA®, Baxalta Innovations, GmbH, Wien, Austria) was added to his treatment protocol. Throughout this period, his inhibitor titer remained consistently negative.

At the age of 7, due to recurrent elbow hemarthroses and a persistently negative inhibitor titer, a re-challenge with rFIX was attempted under close medical supervision. The first two infusions were uneventful; however, the third infusion triggered another anaphylactic reaction, characterized by abdominal pain, respiratory distress, and severe hypotension. His inhibitor titer rose to 0.6 BU. The episode was managed successfully with hydrocortisone, epinephrine, and intravenous fluids. Subsequently, the patient resumed treatment with rFVIIa for episodic bleeding, supplemented with APCC during severe bleeding episodes.

At 8 years old, seven months after the second anaphylactic reaction, a desensitization protocol was initiated. Plasma-derived FIX was gradually introduced, escalating to a dose of 50 IU/kg over five days. After one month, his inhibitor titer remained undetectable, and he transitioned to prophylactic plasma-derived FIX administration twice weekly. This regimen significantly reduced the frequency of bleeding episodes and improved the patient's quality of life.

At the age of 11, the patient developed another elbow hemarthrosis and a significant muscle hematoma in the right wrist extensor, requiring high-dose plasma-derived FIX infusions (56 IU/kg/day for three days, followed by 94 IU/kg/day for two additional days). After 184 cumulative infusions of plasma-derived FIX, he experienced a third anaphylactic reaction, characterized by abdominal pain, dyspnea, cyanosis, and hypotension. His inhibitor titer increased to 3.5 BU, peaking at 7.6 BU two weeks later. This reaction necessitated a return to an on-demand regimen with rFVIIa for the management of recurrent joint bleeds and muscular hematomas.

In May 2019, the patient was enrolled in the Explorer 6 observational study evaluating anti-TFPI therapy, which concluded in November 2019 (14). During this period, he experienced recurrent musculoskeletal bleeding, hemarthroses, and complications such as rectal bleeding, macrohematuria, and mandibular hematoma, resulting in frequent hospitalizations. In December 2019, the patient transitioned to the Explorer 7 trial (funded by Novo Nordisk; Explorer7 ClinicalTrials.gov number, NCT04083781), a randomized study assessing concizumab vs. on-demand therapy (4). He was initially assigned to the on-demand treatment arm. The trial was temporarily paused from March to September 2020 for safety reasons.

On October 13, 2020, the patient initiated concizumab therapy with a loading dose of 1 mg/kg, followed by a daily maintenance dose of 0.2 mg/kg. The initiation of concizumab therapy marked a significant turning point in his management, resulting in a dramatic improvement in bleeding control (results in Table 1 as ABRj, Annualized Bleeding Rate at Joints). Between October 2020 and October 2021, he experienced only two bleeding events. In 2022, there was a single bleeding episode, followed by one subcutaneous hematoma in 2023, which occurred after a syncopal episode. Notably, in 2024, the patient reported no bleeding events at all. Simultaneously, his reliance on rFVIIa decreased significantly, from 516 infusions in 2018 to 11 infusions in 2022, with no further need for rFVIIa thereafter. Importantly, since initiating concizumab therapy, the patient has not required hospitalizations, emergency room visits, or surgical interventions. Joint health assessments using the Hemophilia Early Arthropathy Detection with Ultrasound (HEAD-US) score and the Hemophilia Joint Health Score (HJHS) demonstrated substantial improvements, indicating better joint function and reduced bleeding frequency.

**Table 1:** Outcomes pre and post concizumab treatment initiation, from 2018 up to 2024.

Outcome	Pre-concizumab treatment period			Post-concizumab treatment period			
	2018	2019	January–September 2020	2021 (since October 2020)	2022	2023	2024
ABR	7 (3 right knee, 1 rectal, 1 toe, 1 left iliopsoas, 1 right ankle)	7 (3 right ankle, 2 right elbow, 1 left elbow, 1 left iliopsoas)	5 (1 right knee, 1 left elbow, 2 right elbow, 1 right ankle)	2 (1 right elbow, 1 left ankle)	1 (Right foot instep (trauma)	1 (Subcutaneous hematoma following syncope)	0
FVIIa infusions	516	443	548	12	11	0	0
Hospitalizations	5	4	1	1	0	0	0
Surgery	3	0	0	0	0	0	0
Emergency room visits	3^a^	5	2	0	0	0	0
HEAD-US^b^	22	19	20	18	14	13	13
HJHS	NA	22	NA	18	14	10	10

ABRj, annualized bleeding rate at joints; HEAD-US, hemophilia early arthropathy detection with ultrasound score; HJHS, hemophilia joint health score; NA: not available.

^a^ Central venous catheter.

^b^ Considered sites: right and left elbow, right and left knee, right and left ankle.

Details on outcomes after concizumab treatment initiation up to 2024 are reported in Table 1.

Concizumab was well tolerated, with no reports of severe adverse events, thromboembolic events, or thrombotic microangiopathy. The only observed side effect was mild injection-site erythema, which resolved spontaneously without intervention.

## 3 Discussion

This case emphasizes the complexities of managing severe hemophilia B with inhibitors, particularly in patients at high risk for anaphylaxis and recurrent bleeding episodes. The transition to concizumab therapy demonstrated substantial improvements in both clinical outcomes and quality of life, reflecting observations from pivotal clinical trials and real-world experiences.

The initiation of concizumab therapy resulted in a marked reduction in the patient's annualized bleeding rate (ABR), with only four bleeding episodes reported over four years. This outcome aligns closely with findings from the Explorer 7 trial, which showed that concizumab significantly reduces ABRs across hemophilia subtypes (4, 5). Moreover, the patient's joint health, assessed using the Hemophilia Joint Health Score (HJHS) and Hemophilia Early Arthropathy Detection with Ultrasound (HEAD-US) score, showed significant improvement. These outcomes reinforce concizumab's efficacy in preventing joint bleeds and preserving joint function, as documented in phase 2 and 3 studies (4, 5).

Concizumab therapy also significantly reduced the patient's reliance on recombinant activated factor VII (rFVIIa) and eliminated the need for hospitalizations, emergency interventions, or surgical procedures. The convenience of its daily subcutaneous administration alleviated the logistical and physical challenges associated with intravenous treatments, contributing to improved treatment adherence and enhanced health-related quality of life (HRQoL). This aligns with previous reviews emphasizing the importance of patient-centric therapies in improving both clinical outcomes and psychosocial well-being (4, 13).

Before the enrolment in Explorer7 clinical trial, the absence of an adequate therapeutical option resulted in chronic arthropathy, physical disabilities, and emotional challenges which over time have significantly affected the patient's quality of life. Therefore, adjunct therapies, such as physiotherapy and pain management, are still needed to optimize further functional outcomes and overall quality of life (1, 5). The social impact of severe hemophilia B also warrants attention. The patient's lack of educational qualifications and employment reflects broader societal challenges faced by individuals with early-onset disabilities.

The psychological burden of hemophilia B, particularly in the context of FIX inhibitors, cannot be overlooked. Concizumab's ability to reduce treatment-related anxiety and enhance HRQoL aligns with patient-reported outcomes from the Explorer 7 trial, which highlighted significant improvements in physical and emotional well-being (4, 5).

Concizumab represents a transformative advance in the management of hemophilia B with inhibitors. Its mechanism of action, targeting tissue factor pathway inhibitor (TFPI), enables effective hemostasis while circumventing the limitations of traditional factor replacement therapies (3, 10). Although further long-term data in real-world context on the effectiveness of anti-TFPI molecules are still needed, our clinical case demonstrates how concizumab therapy was able to improve the clinical outcomes and overall quality of life of our patients providing a renewed sense of normalcy and alleviate treatment burdens. The availability of such therapies in the future could represent the opportunity to reduce patients’ long term joint damages caused by the absence of appropriate therapy, avoid the burden of frequent intravenous injections and reduce the management costs of bypassing therapy, hospitalizations, and arthropathy-related surgeries.

## Data Availability

The original contributions presented in the study are included in the article/Supplementary Material, further inquiries can be directed to the corresponding author.
